# The Interferon-Induced Transmembrane Protein 3 -rs12252 Allele May Predict COVID-19 Severity Among Ethnic Minorities

**DOI:** 10.3389/fgene.2021.692254

**Published:** 2021-08-09

**Authors:** Fahad S. Mohammed, Yahya Nauman Farooqi, Suneel Mohammed

**Affiliations:** ^1^Trinity College of Arts and Sciences, Duke University, Durham, NC, United States; ^2^Department of Biology, Mount Allison University, Sackville, NB, Canada; ^3^Department of Medicine, Carolinas HealthCare System - Blue Ridge, Morganton, NC, United States

**Keywords:** IFITM 3, SARS-CoV-2, COVID-19 severity, IFITM (rs12252), population genetics

## Introduction

The COVID-19 pandemic has infected 176 million people and caused 3.8 million deaths worldwide. Artificial intelligence applications and Genome Wide Association Studies (GWAS) have helped us better understand factors that predict COVID-19 severity (The Severe Covid-19 GWAS Group, 2020). The pandemic has disproportionately affected African Americans, Hispanics, native Americans and other ethnic minorities with higher levels of hospitalization and higher mortality rates (Kirby, 2020). It is crucial to identify genetic alleles predicting severity and mortality among ethnic minorities. Interferon-induced transmembrane proteins (IFITMs) are a family of proteins that inhibit viral entry into host cells. The IFITM3-rs-12252 allele has been associated with severe disease and death with influenza and other viruses. We explore the potential role of this allele on the mortality and severity of illness of patients with Severe Acute Respiratory Syndrome Coronavirus 2 (SARS-CoV-2) infection.

## Evolutionary Data

Microorganisms can become pathogenic resulting in human diseases, epidemics and pandemics. They can also coexist with us as part of our microbiome or becoming integrated into our genome. Human genome has evolved over millions of years of natural selection by positive selection of protective traits and negative selection of deleterious traits (alleles) (Pittman et al., 2016). The earliest allele discovered was the heterozygous sickle-cell trait, that reduced the risk and severity of malaria. Another well-characterized allele is the 32 base pair deletion in the gene chemokine receptor CCR5 gene (CCR5-Δ32). Individuals homozygous for this allele are resistant to HIV and carriers have a reduced risk of infection. The frequency of this allele suggests that it may have conferred resistance against the *Yersinia pestis* and the smallpox virus, which have caused epidemics since prehistoric times.

The deadliest pandemic in modern times was the 1918 Pandemic Flu with approximately 50 million deaths, followed by annual flu outbreaks and pandemics of lesser magnitudes. The IFITM3 rs-12252 allele has been associated with severe Influenza. The frequency of this allele in different populations may impact the outcome of the SARS-COV2 pandemic.

## The IFITM Proteins

The Interferon Inducible transmembrane protein's (IFITMs) are a family of evolutionarily conserved viral restriction proteins which have been shown to inhibit the infection of enveloped RNA viruses from 9 viral families including Influenza, dengue virus, Ebola virus and Coronavirus. IFITM1-3 is the first line defense against pathogen infection in vertebrates (Bailey et al., 2014) and is activated in response to type I and II interferon stimulation. IFITM1 is localized on the plasma membrane and restricts the entry of viruses that enter through the plasma membrane. IFITM2 and 3 are localized to early and late endocytic vesicles and lysosomes and restrict viruses that enter through late endocytic compartments. IFITM5 and IFITM10 have no known human immune functions but IFITM5 has been associated with osteoblast function and mutations have been associated with bone demineralization (Zhytnik et al., 2019). Examination of the primate genomes showed that the IFITM3 gene is the most ancient member of the IFITM locus (Compton et al., 2016) and the most important IFITM in preventing the entry of RNA viruses. Human mutant IFITM1, IFITM2, and IFITM3 were generated by CRISPR-Cas9 gene editing and showed that IFITM3 can involve vesicles bearing entering virus and shuttle the late endocytic vesicles bearing viruses to lysosomes for degradation (Spence et al., 2019).

## The IFITM3-RS-12252 Allele Results in Severe Influenza

Many alleles have been correlated with an increase in the severity of influenza, but the most studied allele is the G variant of the SNP rs12252 of the IFITM3 (IFITM3 rs12252). Knockout mice lacking normal IFITM3 were observed to develop fulminant viral pneumonia when exposed to a low-pathogenicity influenza virus (Everitt et al., 2012). A statistically significant number of hospitalized subjects with H1N1 influenza had the IFITM3 rs12252 allele (Zhang et al., 2013). Individuals with the at-risk IFITM3 rs12252 allele had a 6-fold risk of severe influenza and were associated with early hypercytokinemia (cytokine storm) and increased mortality with influenza infection (Wellington et al., 2019).

## Biological Effect of the Mutant IFITM3-RS-12252 Protein

The IFITM3 gene is predicted to encode a wild-type protein which exists as transmembrane protein. The substitution of A with G in the IFITM3 rs12252 allele was thought to cause aberrant splicing of IFITM3 mRNA. This rs-12252 mutation site encompasses a 20-YEML-23 tetrapeptide that is an endocytic signal essential for endocytosis and localization. Proteins engineered with a Y20 mutation converted IFITM3 from inhibitors to enhancers of Middle East Respiratory Syndrome associated Coronavirus (MERS-CoV) and Severe Acute Respiratory Syndrome associated Coronavirus (SARS-CoV) entry into cells *in vitro* (Zhao et al., 2018) and of SARS-COV2 viral entry (Shi et al., 2021).

## Prevalence of IFITM3-RS-12252 Allele

The prevalence of the IFITM3 rs12252 allele is available from multiple databases^1^ (Table 1). The 1000 Genomes Project (5,008 individuals) showed an allele frequency for the G-variant of 0.528 in the East Asian (Chinese, Japanese, Vietnamese) population, 0.26 in the African population, 0.04 in European population, 0.17 in the Hispanic American population and 0.14 in a South Asian population (The 1000 Genomes Project Consortium, 2015). The gnomAD genomes database (143,048 samples) showed an allele frequency of 0.25 among African Americans, 0.117 among Latino American and 0.039 among Europeans (Karczewski et al., 2020). The NCBI ALFA database (11,176 samples) showed an allele frequency of 0.055 among Europeans and 0.15 among African Americans (Phan et al., 2020). The NHLBI Exome sequencing Project (12,094 samples) showed an allele frequency of 0.22 among African Americans and 0.040 among European Americans (Exome Variant Server, 2021). The allele frequency was 18% in Mestizo (a mixed race of Indigenous and Spanish) populations in Western Mexico (López-Jiménez et al., 2018). With a larger combined sample size of 168,953 the allele frequency of G variant was 0.01 to 0.055 in European and European American population, 0.15 to 0.31 in an African and African American population, 0.18 to 0.21 in a Mexican American population, 0.47 to 0.52 in an East Asian population and 0.10 to 0.16 in a South Asian population.

**Table 1:** Allele Frequency of the IFITM-rs12252 alleles among various populations.

**Database**	**Population**	**Allele frequency A**	**Allele count A**	**Allele frequency G**	**Allele count G**
1000 Genome Project	All	0.764	3,824	0.236	1,184
	**African All**	0.740	978	0.260	344
	African Caribbean	0.740	978	0.26	344
	African American SW USA	0.697	85	0.303	37
	Esan Nigeria	0.788	156	0.212	42
	**Americas All**	0.823	571	0.177	123
	Medellin, Columbia	0.926	174	0.074	14
	Mexican Ancestry, Los Angeles	0.781	100	0.219	28
	**East Asians, All**	0.472	476	0.528	532
	Xishuangabanna, China	0.522	97	0.478	89
	Han, Beijing	0.461	95	0.539	89
	Southern Han, China	0.495	104	0.505	106
	**Europeans All**	0.959	965	0.041	41
	European Ancestry Utah	0.955	189	0.045	9
	Great Britain	0.989	180	0.011	2
	Iberia, Spain	0.967	200	0.033	7
	**South Asian All**	0.853	834	0.147	144
	Bangladesh	0.837	144	0.163	34
	Indian, UK	0.897	183	0.103	34
gnomAD genomes	All	0.873	124,917	0.127	18,131
	African American	0.750	31,451	0.250	10,467
	Latino American	0.883	12,043	0.117	1,597
	Non-Finnish European	0.961	61,988	0.039	2,512
	South Asian	0.867	2,636	0.134	406
NCBI ALFA	European	0.945	7,690	0.055	444
	African American	0.849	569	0.151	100
NHLBI Exome Sequencing Project	African American	0.771	3,005	0.229	891
	European American	0/960	7,955	0.040	333

## Correlation of the IFITM3-RS-12252 Allele With SARS-COV-2 Severity and Mortality

In a study of hospitalized patient with COVID-19 in Beijing, China there was a significant association between homozygosity for the IFITM3-rs12252 allele and severity of COVID-19 and death (Zhang et al., 2020). The frequency of the homozygosity for the IFITM3-rs-12252 in patients with mild disease was similar to the general population. This allele was also associated with a risk of hospitalization in a cohort of hospitalized patients with COVID-19 in Spain (Gómez et al., 2021). This cohort was a Caucasian population with a low frequency (0.03) of the IFITM3-rs-12252 allele. This allele was also non-significantly more common in patients requiring intensive care. Another allele of the IFITM3 rs34481144 has been associated with severity of influenza. A significant correlation was observed between the mortality rate with COVID-19 for ethnic groups in Britain and the combined frequency of IFITM3 alleles rs12252 and rs34481144 in these ethnic groups, suggesting that these alleles may be associated with more severe outcomes (Nikoloudis et al., 2020). The IFITM3-rs12252 variant was also associated with hospitalization and mortality in a study of 880 patients from Saudi Arabia, and they had a lower level of Interferon Gamma (Alghamdi et al., 2021). In contrast, a study from Germany did not find an association with infection risk or severity but suggested that this allele may be important for predicting infection risk (Schönfelder et al., 2021).

While socioeconomic factors and disparities in access to healthcare may partly explain the disproportionately higher mortality and morbidity associated with SARS-COV-2 infection among African Americans, genetic factors may also be responsible for the wide differences seen. The higher frequency of the IFITM3-rs-12252 allele among African Americans (0.15–030) compared to Caucasians (0.01–0.05) may also be an important factor in the higher severity and death rate seen with COVID-19 in African Americans.

An analysis of publicly available transcriptomic data sets of SARS-COV-2 infected humans and mammals identified IFITM3 as an early upregulated gene in infected lung epithelial cells (Hachim et al., 2020). Gene expression analysis of host human genes induced by SARS-COV-2 and SARS-COV shows that IFITM-3 was upregulated by SARS-COV-2 but not SARS-COV (Sardar et al., 2020). This suggests that SARS-COV-2 uses IFITM-3 for viral entry, and that mutations that enhance viral entry may lead to a severe disease. The IFITM3-rs12252 allele involves a four amino-acid tetrapeptide endocytic signal. Artificial mutations of this endocytosis-promoting Yxxϕ motif converts IFITM3 from an inhibitor to an enhancer of and of SARS-COV2 viral entry (Shi et al., 2021).

## Discussion

Currently, the Covid-19 pandemic has resulted in 34 million cases and 615,000 deaths in the United States. The mortality has disproportionally affected African American, Hispanic, and Native American populations. This disparity may be because African Americans have higher incidence rates of obesity and diabetes and often hold jobs that have an increased risk of community exposure (Price-Haywood et al., 2020). A disproportionate incidence of COVID-19 infections, hospitalizations and deaths have also been noted in the Hispanic population in the United States (Evans, 2020). American Indians and Alaska Natives were 3.7 times more likely to be hospitalized and 2.4 times more likely to die compared to non-Hispanic whites in the United States (Burki, 2021). In Britain, the worst outcomes are in ethnic minority groups, especially Black and South Asian people (Razai et al., 2021).

Socioeconomic factors, disparity in access to health care and structural racism are important factors in the severity and mortality rate among ethnic minority Covid-19 patients (Kirby, 2020). But this cannot fully account for the disproportionately higher morbidity and mortality among ethnic minorities and genetic factors may be important. The IFITM3 is important in the first line of defense against SARS-COV-2, and alleles like the IFITM3-rs-12252 may convert it from an inhibitor to an enhancer of SARS-COV-2 viral entry to cells. The altered viral endocytosis in patients with the IFITM3-rs12252 allele may causes high viral load and cytokine storm associated with severe Covid-19 in susceptible patients. The higher frequency of the at-risk IFITM3 rs12252 allele among African Americans (0.25), Hispanics (0.20), and South Asians (0.15) compared to European ancestry (0.01–0.04) may contribute to the disproportionately higher morbidity and mortality among ethnic minorities.

SARS-COV-2 is a mutated cold virus which caused mild illness in most patients. Viruses become highly pathogenic by selecting for specific host genetic variants within the target population. SARS-COV-2 causes severe morbidity and mortality only in certain population subtypes and genotypes. We are suggesting that SARS-COV-2 infections can results in severe illness and death in individuals and populations with the IFITM3-rs12252 G variant. Modern medicine, epidemiological measures, vaccination, isolation and containment efforts can counteract the negative evolutionary pressures on deleterious mutations in the twenty first century. An example of this is the lower morbidity and mortality from COVID-19 seen in China. Despite a high allele frequency of 0.50 of the at-risk IFITM3-rs-12252 allele among the Chinese population, the mortality and morbidity were low in China in the first 3 months of the outbreak excluding Wuhan (Liu et al., 2021). The lead author of the study attributes the low mortality in China to the lockdown and associate behavioral changes like facemasks, increased hygiene, social distancing and travel restrictions.

It was recently reported that the antibody response to the Influenza vaccination was attenuated in IFITM3-rs-12252 homozygous carriers and in IFITM3 deletion mice (Lei et al., 2020). Since COVID-19 vaccination efforts are well underway in most of the world, the antibody response to COVID-19 vaccination in IFITM3-rs-12252 allele carriers needs to be studied and corrective measures undertaken if needed.

The COVID-19 pandemic is waning in the Western countries while it is making a resurgence in India and Africa. We are still early in the pandemic and the SARS-COV-2 virus may mutate to more pathogenic variants and cause annual epidemics. The SARS-COV-2 virus B.1.617.2 (Delta variant) is projected to be the dominant variant in the USA and UK soon (Paul et al., 2021). The delta variant is highly contagious. We should not let our guard down and continue containment measures albeit in a smarter, and less intrusive way and institute early intensive medical care for patients at higher risk of severity. These should be focused on vulnerable ethnic minorities. The role of the IFITM3-rs12252 allele in the higher morbidity and mortality from COVID-19 among African American and other ethnic minorities should be studied and better understood. Understanding the interaction between SARS-CoV-2 and the IFITM3-rs12252 allele could help identify novel therapeutic targets, leading to precise biological and immune therapy.

## Author Contributions

All authors certify that they have participated sufficiently in the work to take public responsibility for the content, including participation in the concept, design, analysis, writing, or revision of the manuscript.

## Conflict of Interest

The authors declare that the research was conducted in the absence of any commercial or financial relationships that could be construed as a potential conflict of interest.

## Publisher's Note

All claims expressed in this article are solely those of the authors and do not necessarily represent those of their affiliated organizations, or those of the publisher, the editors and the reviewers. Any product that may be evaluated in this article, or claim that may be made by its manufacturer, is not guaranteed or endorsed by the publisher.

## Footnotes

^1^Available online at: http://useast.ensembl.org/Homo_sapiens/Variation/Population?db=core;r=11,320272-321272;v=rs12252;vdb=variation;vf=164428450#population_freq_AFR.
